# Retraction Note: Transdermal delivery of allopurinol-loaded nanostructured lipid carrier in the treatment of gout

**DOI:** 10.1186/s40360-023-00680-z

**Published:** 2023-07-12

**Authors:** Zakir Ali, Fakhar ud Din, Fatima Zahid, Saba Sohail, Basalat Imran, Salman Khan, Maimoona Malik, Alam Zeb, Gul Majid Khan

**Affiliations:** 1 Nanomedicine Research Group, Department of Pharmacy, Quaid-I-Azam University, Islamabad, Pakistan; 2 Department of Pharmacy, Faculty of Biological Sciences, Quaid-I-Azam University, Islamabad, Pakistan; 3 Department of Pharmacy, Riphah International University, Islamabad, Pakistan; 4 Islamia College University, Peshawar, Pakistan

**Retraction Note: BMC Pharmacology and Toxicology (2022) 23:86**

10.1186/s40360-022-00625-y

The Editor has retracted this article. After publication, concerns were raised regarding image similarities in Figs. 5 and 8. Specifically:

In Fig. 5a and b, images representing groups II and III appear to originate from the same animal;

In Fig. 8, all images appear to originate from two samples with slight modifications.

The authors have provided the raw data to address these concerns; however, the publisher could not validate the authenticity of these data. The Editor therefore no longer has confidence in the presented data and the conclusions of this article.

Zakir Ali, Fatima Zahid, Maimoona Malik and Saba Sohail agree to this retraction. Fakhar ud Din has agreed to this retraction but not to the wording of this retraction notice. Basalat Imran, Salman Khan, Alam Zeb and Gul Majid Khan have not responded to any correspondence from the editor or publisher about this retraction.

